# A new method of recording attendance improves the academic performance of medical students

**DOI:** 10.30476/jamp.2020.81723.1029

**Published:** 2020-04

**Authors:** HIMEL MONDAL, KOUSHIK SAHA, SHAIKAT MONDAL, PIYALI SAHA, SAIRAVI KIRAN BIRI

**Affiliations:** 1 Department of Physiology, Bhima Bhoi Medical College & Hospital, Balangir, Odisha, India; 2 Department of Anatomy, Rampurhat Government Medical College and Hospital, West Bengal, India; 3 Department of Physiology, Raiganj Government Medical College and Hospital, West Bengal, India; 4 Department of Special Education, Netaji Subhas Open University, Kolkata, West Bengal, India; 5 Department of Biochemistry, Fakir Mohan Medical College and Hospital, Balasore, Odisha, India

**Keywords:** Academic performance, Attention, Medical education, Students

## Abstract

**Introduction:**

Students’ engagement during the collection of attendance (SEdCA) is a method where students write the answer to a question related to the topic of preceding 1-h lecture. Then, attendance is recorded by the teacher from the answer sheets. This method was introduced primarily to overcome difficulty in recording attendance from a class of high attendance. Its potential formative assessment capability has not yet been ascertained. With this background, the aim of this study was to evaluate the effect of the application of SEdCA as a method of formative assessment on the academic performance of first-year medical students.

**Methods:**

This interventional, uncontrolled, before and after study was conducted on 93 first-year medical students. Part completion test (PCT) scores in anatomy before the application of SEdCA was considered as the pre-intervention academic performance. Then, 1-h lectures were designed according to SEdCA for a period of 3 months. The next PCT scores were taken as post-intervention performance and compared with the pre-intervention performance using paired t-test with α = 0.05.

**Results:**

Ninety-three (female=38, male=55) first-year medical students with a mean age of 17.65±0.88 years participated in the study. There was a significant increase in theory (23.74±5.67 versus 26.40±5.17, *t*=3.31, *P*<0.001), practical (21.43±6.60 versus 24.08±5.16, *t*=6.95, *P*<0.001), and total (45.17±11 versus 50.47±9.17, *t*=8, *P*<0.001) scores in the post-intervention PCT.

**Conclusion:**

SEdCA may be applied to enhance the academic competency of first-year medical students. However, its impact should be evaluated further in multiple subjects in students of different years of study in more institutes for a generalized result.

## Introduction

In India, medical colleges have different annual intake capacity for admitting undergraduate student ranging from 50-250 ( 1 ). Small group teaching or small group discussion helps the learners to improve their knowledge, attitudes, and skill ( 2 ). Small group teaching is better not only for basic sciences, but also for learning evidence-based medicine ( 3 ). Faculty and students both support the advantage of small group teachings ( 4 , 5 ). However, developing countries like India are faced with shortage of medical teachers ( 6 ). Hence, teachers or the institutions often have no option but to conduct lecture classes with as high as 250 students. For a small group of students, it is not difficult to record attendance by traditional roll call method or digital methods ( 7 ). In contrast, collection of attendance from a class with high attendance (e.g., 250) is a tedious and time-consuming job requiring approximately more than 10% of the 1-h lecture. In some instances, for recording attendance, we compromise the time of teaching. Hence, traditional roll call method causes a loss of huge academic hours annually as the students commonly sit idle during the roll call. If these hours could have been utilized for formative assessment, it could increase the academic competency of the students.

For solving this emerging problem of the collection of attendance from a class with high attendance, a new method of recording attendance has been introduced. Students’ engagement during collection of attendance (SEdCA) is the method where teachers ask students to write the answer to a question with the name and roll number. These answer sheets are collected by the teachers and scrutinized for roll numbers for recording attendance later in any convenient time of the teacher ( 8 ). Though this method was introduced for a class of 250 students, its utility in recording attendance and simultaneously conducting a formative assessment can be adopted in a class of any number of students.

Formative assessment helps the students to enhance their learning ( 9 ). Frequent conduct of formative assessment creates an academic environment where students learn a small part of the course effectively rather than a high volume before the summative assessment ( 10 ). Previous studies showed that formative assessment helps students in the improvement of the academic performance in medical, allied health, and non-medical students ( 11 - 14 ). The improvement is also reflected in the summative assessment ( 15 ). SEdCA can serve both as a way of a collection of attendance as well as a method of formative assessment. This assessment would reflect the learning of a student from the preceding 1-h lecture.

With this background, our research question was about the applicability of SEdCA as a method of formative assessment in the improvement of academic performance in medical students. For finding the answer to this research question, the objective of this study was set to find academic performance before and after application of SEdCA and to compare those in medical students. The finding of this study would help the teachers and institutions in informed adaptation of this new method of recording attendance as a tool for formative assessment.

## Methods

### *Type and settings*

This study was an uncontrolled, before and after study where we used an intervention on the whole sample. We evaluated the effect of the intervention by comparing the outcome before and after the intervention ( 16 ). The study was conducted in a government-aided medical college situated in Eastern India for a period of 3 months in the year 2019.

### *Samples and sampling method*

A convenient sample was used for this study with the inclusion criteria being any first-year medical student and providing written consent for participation. There was no exclusion criterion. Students were first briefed about the aim and method of the study and the students willing to participate were asked to sign the informed consent form. They were assured that their anonymity would be maintained throughout the study. Among 99 students of the class, one student was absent from the beginning of the course. A total of 5 students could not be contacted for briefing about the study due to absence in multiple sessions; hence, they were excluded from the study. The final sample size was 93 of first-year medical students.

### *Pre-intervention academic performance assessment*

Part completion test (PCT) or part completion examination is a method of summative assessment after completion of an anatomical segment of the human body (e.g. the superior extremity, abdomen) ( 17 ). This method of internal assessment is carried out for first year medical students who study anatomy, physiology, and biochemistry for a span of 1 year. Scores in the PCT of anatomy were considered as the indicator of academic performance for this study. Both theory and practical marks of the PCT were obtained from the departmental result register.

### *Intervention*

1-h lectures were designed with SEdCA so that the last 8-10 minutes of 1-h was left for execution of the method. At the beginning of the lecture, students were briefed about the method and informed that this method would be used for collection of attendance at the end of the lecture. According to the original method of SEdCA, students were asked questions on the topic taught in the lecture. Students took a spare sheet from their exercise book and wrote the answers with their roll numbers. The answer sheets were collected and checked by a single teacher for evaluation and recording attendance. A total of 44 1-h lecture was conducted with SEdCA.

### *Post-intervention academic performance assessment*

The theory and practical marks of the very next anatomy PCT was considered as the post-intervention summative assessment scores. In both pre- and post-intervention phase, the theory question was objective and one-liner type and the practical one was objective structured. This type of questions reduced any assessors’ bias in the obtained scores.

### *Statistical analysis*

Data were expressed in mean and standard deviation and pre-intervention and post-intervention data were compared by paired t-test. Data of male and female students were compared by independent sample t-test. Pearson correlation was calculated for pre- versus post-intervention scores. Increase, unchanged, or decreased scores in the pots-intervention PCT was expressed in numbers only (no percentage was calculated) as the sample size was below 100. Change in the scores (post-intervention marks – pre-intervention marks) was plotted against pre-intervention cores for a visual representation of the increment, unchanged and decrement of the marks. Statistical analysis was carried out on GraphPad Prism 6.01 (GraphPad Software, Inc., CA, USA).

## Results

Ninety three (female = 38, male = 55) first year medical students aged 17.65±0.88 (male = 17.74±0.92, female = 17.58±0.85, independent sample *t* = 0.83, *P* = 0.41) years participated in this study.

Sex-wise pre- and post-intervention of theory, practical, and total marks are shown in Table 1. There was a statistically significant increase in theory, practical, and total scores in post-intervention summative assessment in both male and female participants.

**Table1:** Pre- and post-intervention marks

	Statistics	Theory			Practical			Total		
		Pre-intervention	Post-intervention	Paired t test P	Pre-intervention	Post-intervention	Paired t test P	Pre-intervention	Post-intervention	Paired t test P
Female (n = 38)	Mean ± SD	23.89 ± 5.5	26.55 ± 4.95	<0.001	20.42 ± 5.68	23.58 ± 4.68	<0.001	44.32 ± 10.26	50.13 ± 8.315	<0.001
	Range	8 - 31	10 - 33		8 - 28	14 - 32		21 - 58	25 - 63	
Male (n = 55)	Mean ± SD	23.64 ± 5.84	26.29 ± 5.36	<0.001	22.13 ± 7.14	24.42 ± 5.48	<0.001	45.76 ± 12.12	50.71 ± 9.79	<0.001
	Range	10 - 33	16 - 38		9 - 36	12 - 36		21 - 68	30 - 68	
Unpaired *t* test P*	0.83	0.81	-	0.22	0.44	-	0.55	0.77	-
Overall (n = 93)	Mean ± SD	23.74 ± 5.67	26.40 ± 5.17	<0.001	21.43 ± 6.6	24.08 ± 5.16	<0.001	45.17 ± 11.36	50.47 ± 9.17	<0.001
	Range	8 - 33	10 - 38		8 - 36	12 - 36		21 - 68	25 - 68	

SD: standard deviation

* Independent sample/unpaired t test was done to compare marks of female and male students

When we compared sex-wise data by independent sample t-test, both in the pre-intervention and post-intervention phase, there was no gender difference in the scores (Table 1).

The correlation coefficient between pre- and post-intervention is shown in Table 2. Pre-intervention theory, practical, and overall scores showed a positive correlation with post-intervention ones. The overall coefficient of determination (R^2^) indicates that we can predict an improvement in 68% of cases. 

**Table2:** Correlation of pre-intervention and post-interventionscores

	Theory	Practical	Overall
r	0.72	0.83	0.83
R^2^	0.52	0.69	0.68
P	<0.0001*	<0.0001*	<0.0001*

r: Pearson correlation coefficient, R2: Coefficient of determination

* Statistically significant correlation

**Figure 1:**
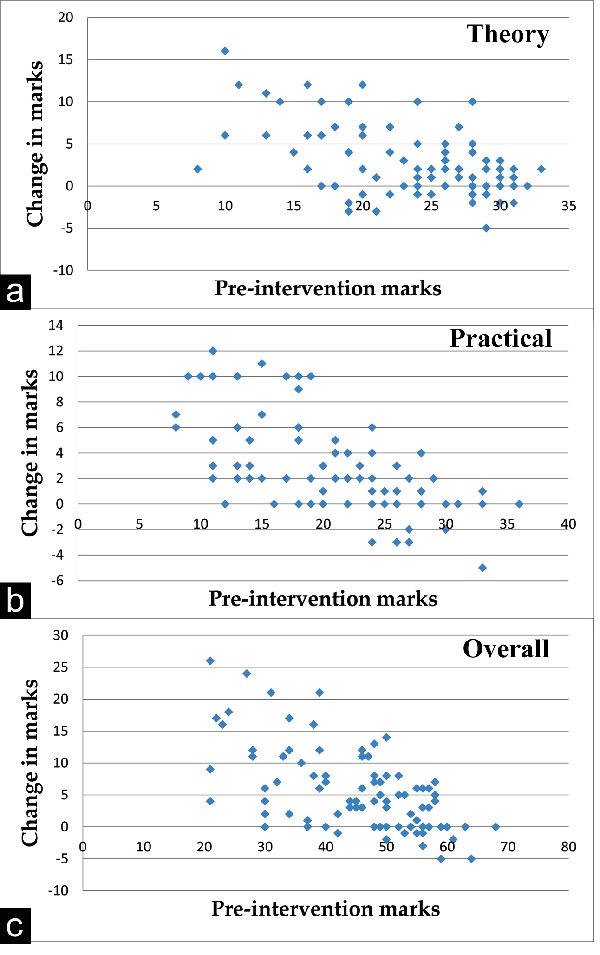
Scatterplot of the pre-intervention scores versus changes in the scores in (a) theory (b) practical and (c) overall subject

In theory, there was an increase in the scores in 59 students, 18 were unchanged, and 16 showed decreased scores. In the practical scores, 62 showed increment, 23 was unchanged, and 8 showed decrement. Overall, there was an increase in 63 students, 20 were unchanged, and 10 showed decrement. The change in the scores (post-intervention marks – pre-intervention marks) was plotted against the pre-intervention scores and is shown in Figures 1a, b, and c.

Footnote: A positive sign indicates increase in scores in the post-intervention test.

## Discussion

### *Formative assessment and its advantage*

Summative assessment is the method of testing students about their learning at a particular interval. In contrast, formative assessment is a continuous evaluation process which is an integral part of the learning ( 18 ). Among the different evaluation methods in medical education, formative assessment is considered as one of the sustainable and promising approaches ( 19 ). The major advantage of the formative assessment over summative assessment is the feedback of the learning. Students can easily evaluate their learning on the particular topic ( 20 , 21 ). In addition, online formative assessment is also found to reinforce self-directed learning ( 22 ). Frequent formative assessment has a positive impact on academic performance ( 23 , 24 ). Competency-based, student-centred teaching-learning environment with early clinical exposure has been a new landscape for Indian medical education ( 25 - 27 ). In this method of education, formative assessment is an integral part. However, as a new method to implement, teachers need to adapt themselves quickly with the method of formative assessment ( 28 , 29 ).

### *Advantages of SEdCA*

The method of SEdCA is simple and does not need any extra financial support from the institutions. This method can be applied at the end of each didactic lecture as a formative assessment method. Even the students can assess themselves or get feedback immediately about their learning in the preceding lecture topic ( 30 ). In addition, the teacher gets a voice rest from calling the rolls ( 8 ).

### *Disadvantage of SEdCA*

Perhaps each new educational method has some limitations in its initial stage. The method gets eventual modifications for becoming a more acceptable one. The method of SEdCA also has a major limitation that the teachers need to invest a huge time in scrutinizing the roll numbers from the sheets submitted by the students. For the formative assessment, only short answer type or multiple choice questions can be asked as the time for writing the answer is limited to the last 8-10 minutes of the 1-h lecture. Additionally, in institutions where the students sit very closely in the classroom, there are chances of cheating. And if this happens, the aim of formative assessment and self-evaluation fails.

### *Outcome of this study*

The major finding of this study is the significant improvement in academic performance after application of SEdCA as a method of formative assessment. Hence, it can be considered as a method of formative assessment along with its advantage of attendance recording. The potential reason for better performance in post-intervention PCT may be due to increased attention in lectures. As the students were well aware that a formative assessment would be conducted at the end of the class, this may make them more attentive. However, this assumption is purely hypothetical and we did not explore this in this study. This topic would be studied in any future research.

### *Gender difference in academic performance*

A sample of first-year medical students with 40.86% female and 59.14% male students showed that there was no gender difference in academic performance both in pre- and post-intervention phase. This finding is corroborative with those of other studies conducted in India and other countries ( 31 - 34 ).

### *Critical evaluation of the finding*

The coefficient of determination showed that in the majority of students (68%) the improvement of academic performance is predictable. However, 32% of the variation is attributed to other factors than the intervention ( 35 ). Further analysis showed that 20 among 93 showed no increase in their scores; even 10 among 93 showed decreased scores in post-intervention PCT. Hence, teachers and educators may pilot the effect of SEdCA on academic performance in their institution before application.

### *Strength and Limitation of the study*

This study first reports an alternative method of recording attendance (i.e. SEdCA) as a way of formative assessment. This can be used in each 1-h lecture where students are engaged in assessing their learning from the preceding class.

Summative assessment of a single pre-clinical subject was considered as an indicator of academic performance. Hence, its impact on multiple subjects is yet unknown. The study carried out in a single government-aided medical college further limits the results for generalization. In addition, we only compared the pre-intervention and post-intervention scores. We did not carry out any case-control study which could provide more acceptable result. However, it was beyond our teaching method as we conduct 1-h lecture of a whole class at a time and have no opportunity to divide them in separate control and case groups. Further research may be done in other institutions using the case-control method.

## Conclusion

For enhancing the academic performance of the first-year medical students, SEdCA is a new method of formative assessment. This method was introduced for overcoming the hurdle of collection of attendance from a large group of students and our study established its formative assessment wing. Hence, it can be adopted in any institution due to its simplicity of application and its dual advantage. However, its impact should be evaluated further in multiple subjects with students in different years of their study in more institutes for a more generalized result.
